# Spectralis OCT1 versus OCT2: Time Efficiency and Image Quality of Retinal Nerve Fiber Layer Thickness and Bruch's Membrane Opening Analysis for Glaucoma Patients

**DOI:** 10.5005/jp-journals-10078-1244

**Published:** 2019

**Authors:** Fabian Bosche, Jil Andresen, Daniel Li, Frank Holz, Christian Brinkmann

**Affiliations:** 1–5 Department of Ophthalmology, University of Bonn, Bonn, Germany

**Keywords:** Bruch's membrane opening, Glaucoma, Optical coherence tomography, Retinal nerve fiber layer, Spectralis

## Abstract

**Purpose:**

To compare two generations of Heidelberg SPECTRALIS optical coherence tomography (OCT) technologies (SPECTRALIS OCT1 and OCT2) with regard to time efficiency and image quality of retinal nerve fiber layer (RNFL) thickness and Bruch's membrane opening (BMO) analysis in individuals with glaucoma.

**Materials and methods:**

In this single center, prospective cohort study, 35 consecutive glaucoma patients (70 eyes) were included. RNFL thickness and BMO-MRW analysis was performed in all patients using the Heidelberg SPECTRALIS OCT1 and the Heidelberg SPECTRALIS OCT2 module. Each patient was imaged three times both with the SPECTRALIS-OCT1 and the SPECTRALIS-OCT2 device. All scans were assessed for further analyzability. Acquisition duration, signal-to-noise ratio (SNR), and the displacement between the initially localized and the redetermined BMO center were extracted from the measurement protocols and statistically compared.

**Results:**

Mean (cumulative) scan acquisition duration was significantly higher with OCT1 compared with OCT2 (54.80 ± 18.61 seconds vs 20.40 ± 6.61 seconds; *p* < 0.01). Patient-related comparison showed a lower scan duration with the OCT2 device in all 35 patients. Mean SNR of the OCT1 images was 29.9 dB and 32.3 dB for the OCT2 images. The difference of −2.4 (95% CI: −3.1 to 2) was highly significant (*p* < 0.001). Mean displacement of the OCT1 images was 42.9 μm and 40.2 μm for the OCT2 images (95% CI: −4.710; *p* = 0.479).

**Conclusion:**

With SPECTRALIS OCT2, acquisition time of BMO and RNFL scans is less than half of the acquisition time of SPECTRALIS OCT1. Image quality of OCT2 module is at least equivalent to the image quality of OCT1.

**How to cite this article:**

Bosche F, Andresen J, *et al.* Spectralis OCT1 versus OCT2: Time Efficiency and Image Quality of Retinal Nerve Fiber Layer Thickness and Bruch's Membrane Opening Analysis for Glaucoma Patients. J Curr Glaucoma Pract 2019;13(1):16–20.

## INTRODUCTION

Besides clinical examination and visual field testing, several imaging technologies have become an essential part of glaucoma diagnostics. Best evaluated and developed instruments are confocal scanning laser ophthalmoscopy (cSLO) like Heidelberg retina tomograph (HRT) and scanning laser polarimetry (SLP) like GDx VCC (variable cornea compensation) and OCT.^1^ Typically used parameters to assess glaucomatous damage by OCT include circumpapillary retinal nerve fiber layer (cpRNFL) thickness and Bruch's membrane opening-minimum rim width (BMO-MRW). These parameters show close correlation to functional parameters such as visual field.^2^ They have been demonstrated as valid parameters for glaucoma detection and glaucoma progression analysis.^3,4^

Optical coherence tomography technology is rapidly evolving. In the 1990s, time domain (TD)-OCT was introduced and showed high diagnostic sensitivity and specificity in several studies.^5–7^ A recent development is spectral domain (SD)-OCT which has a higher axial resolution and a higher scanning speed than TD-OCT.^8–10^ Heidelberg Engineering offers two generations of SD-OCT devices, Spectralis OCT1 and Spectralis OCT2. For the newer Spectralis OCT2 module, the spectrometer has been redesigned. It includes a line scan camera with an A-scan rate of 85 kHz. The previous Spectralis OCT1 module includes a line scan camera with an A-scan rate of 40 kHz. The higher A-scan rate of OCT2 results in an increased acquisition speed at the same sampling density. Herein, we compared the first and the second generation of the Spectralis SD-OCT devices (OCT1 and OCT2) with regard to speed and image quality of RNFL and BMO analysis.

## MATERIALS AND METHODS

In this randomized, single-center study, 35 patients with manifest glaucoma were recruited from the glaucoma clinic at the Department of Ophthalmology, University of Bonn, Germany. Inclusion criteria were age >18 years, clear ocular media, and open angle glaucoma. Examination was performed by trained photographers. Informed consent was obtained.

**Figs 1A and B:**
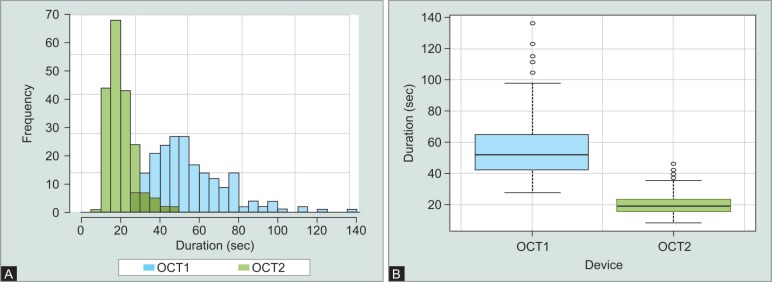
Comparison of acquisition time of OCT1 and OCT2. (A) Acquisition duration distribution for OCT1 (blue) and OCT2 (green) in the study group (*n* = 34); (B) Mean acquisition duration of OCT1 (blue) and OCT 2 (green) in the study group

The Heidelberg Spectralis OCT1 module and the Heidelberg Spectralis OCT2 module were used for imaging. The acquisition sequence (OCT1–OCT2 or OCT2–OCT1) for each patient was randomized. Three complete scans per eye were recorded both with the Spectralis OCT1 and the Spectralis OCT2 device on the same day. The total number of scans was 420. Each complete scan consisted of 24 equidistant radial scans and 3 concentric circle scans of 3.5 mm, 4.1 mm, and 4.7 mm diameter, which were all placed at the BMO center at the optic nerve head (ONH). The minimum rim width (MRW), defined as the minimum distance between the BMO and the internal limiting membrane (ILM), was automatically determined in all of the 24 radial scans (Fig. 1).

RNFL thickness was measured in the 3 circle scans (see Fig. 1B). Fifty-one complete scans were excluded for different reasons (mostly poor image quality). A total of 369 scans of 62 eyes in 34 patients were included in statistical analysis. Scan duration could be determined on the basis of the acquisition protocols. Signal-to-noise ratio was extracted from standard image information given in the SPECTRALIS software.

For precision analysis, we used the displacement between the automatically localized BMO center (by anatomic positioning system; APS) and the redetermined center of BMO which is calculated from the 27 radial and circle scans. A normal distribution of the measurement data was checked by histograms. Acquisition duration, SNR, and BMO center displacement of the two OCT devices were compared by unpaired *t* test. Univariate regression analysis was used for correlations between patient age and acquisition duration. Statistical analysis was performed with R software.

## RESULTS

**Fig. 2:**
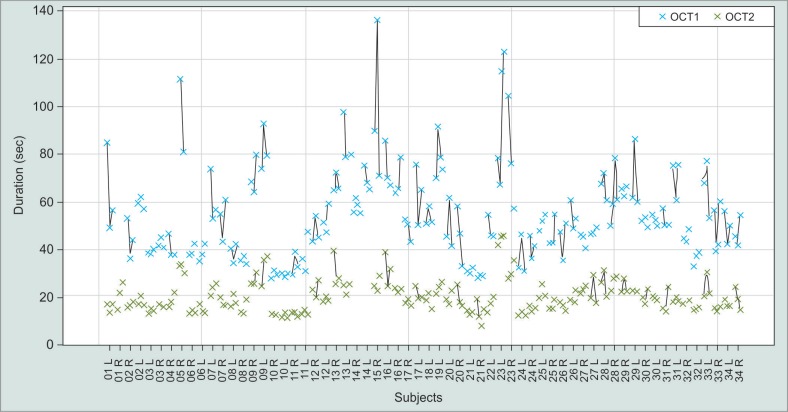
Patient specific acquisition duration of OCT1 (blue) and OCT2 (green). Crosses symbolize complete scans (three concentric circle scans and 24 radial scans). Numbers stand for different patients. L = left eye, R = right eye

In our study group (*n* = 34), the mean scan duration was 54.80 ± 18.61 seconds for OCT1 and 20.40 ± 6.61 seconds for OCT2 (Fig. 1). The difference of 34.40 seconds was highly significant (*p* < 0.01). Patient-specific comparison shows a shorter scan duration of OCT2 and less variation between the three repeated scans throughout the whole study group (Fig. 2).

**Figs 3A and B:**
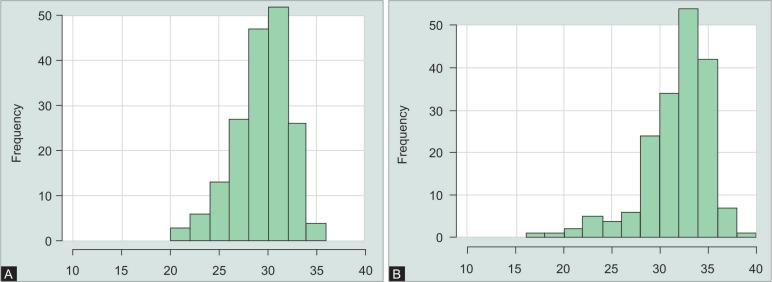
Distribution of signal-to-noise-ratio of the Bruch's membrane opening-scans performed with (A) OCT1 and (B) OCT2

**Figs 4A and B:**
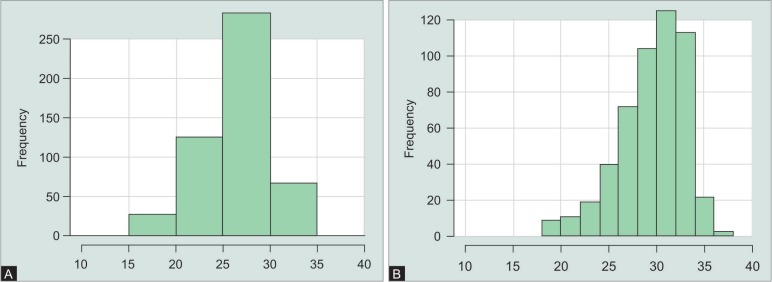
Distribution of signal-to-noise-ratio of the circumpapillary retinal nerve fiber layer scans performed with (A) OCT1 and (B) OCT2

**Figs 5A and B:**
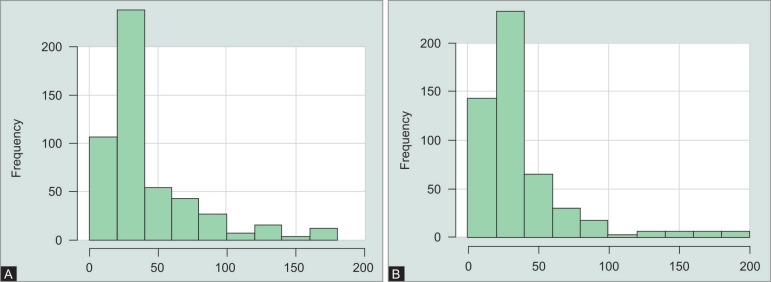
Distribution of displacement of the circumpapillary retinal nerve fiber layer RNFL-Scans performed with (A) OCT1 and (B) OCT2

The mean SNR of the BMO scans with (Fig. 3) OCT1 was 29.9 dB and 32.3 dB for the BMO scans performed with OCT2 (Fig. 4). The difference of −2.4 (95% CI: −3.1 to 2) was significant (*p* < 0.001). The mean SNR of the RNFL scans performed with OCT1 was 26.6 dB and 30 dB for the RNFL scans performed with OCT2 (Fig. 5). The difference of −3.4 (95% CI: −3.8 to 3) was also significant (*p* < 0.001).

**Fig. 6:**
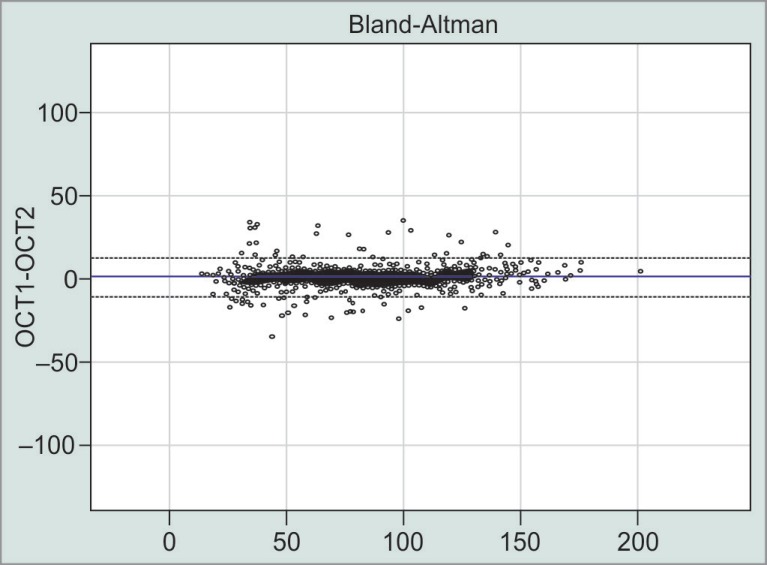
Differences (in micrometers) between OCT1 and OCT2 for all circumpapillary retinal nerve fiber layer scans and (circles). Range of the mean values is shown on *X*-axis

The mean displacement between the initially localized and the redetermined BMO center was 42.9 μm with OCT1 and 40.2 μm for scans performed with OCT2 (Fig. 6). The difference of 2.7 (95% CI: −4.710) was not significant (*p* = 0.479).

**Figs 7A and B:**
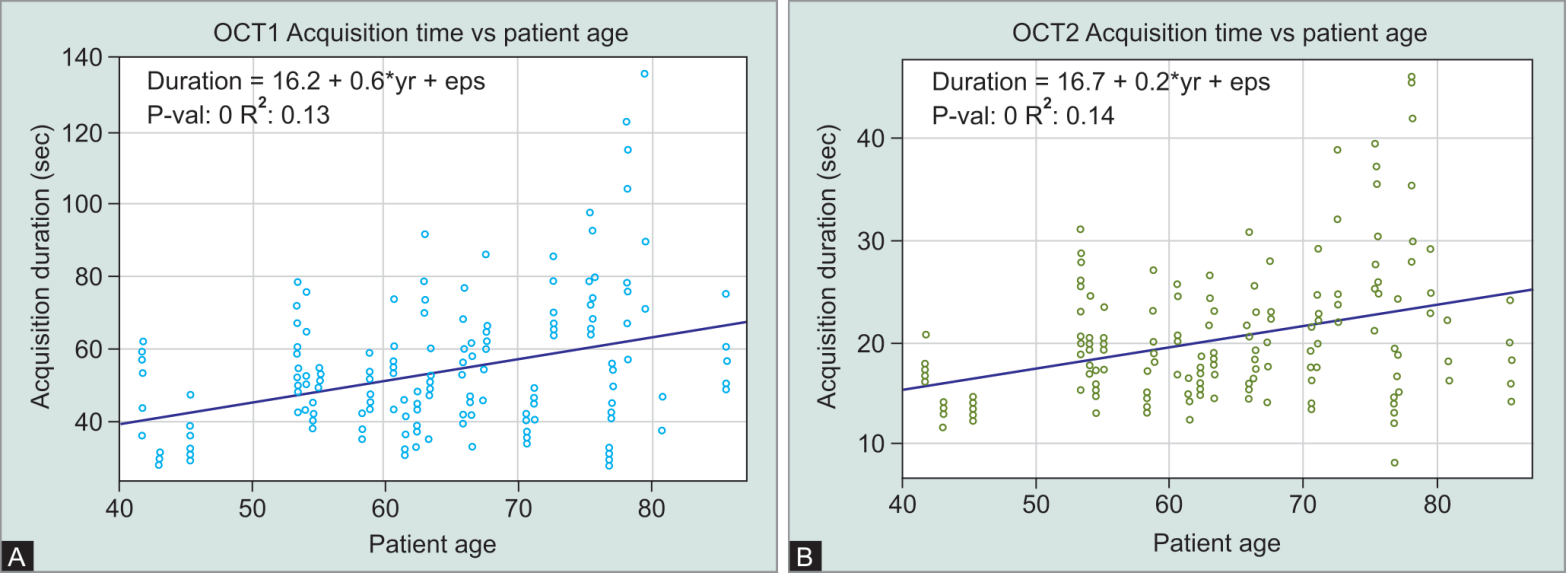
Linear regression of patient age versus acquisition duration with (A) OCT1 and (B) OCT2

Univariate linear regression analysis showed a significant correlation between the patient age and the acquisition duration for both OCT1 and OCT2 devices (Fig. 7). The variation of scan duration markedly increased with patient age (Fig. 7).

## DISCUSSION

Herein we compared Spectralis OCT1 vs OCT2 technology with regard to acquisition duration and image quality of BMO and RNFL analysis. To our knowledge, this is the first study that investigates the performance of OCT2 technology in glaucoma diagnostics.

The results demonstrate a significant shorter acquisition time and a better SNR with Spectralis OCT2. The striking difference of the mean acquisition durations (OCT2: 20.40 ± 6.61 seconds, OCT1: 54.80 ± 18.61 seconds) is based on the higher A-scan rate (85 kHz) of the newly designed spectrometer of the OCT2 device. Other studies comparing macula scans of OCT1 and OCT2 found acquisition time savings of approximately 25% with Spectralis OCT2. Shorter acquisition time goes along with less fatigue for the patient. This could explain the lower variability between the single examinations of each patient with OCT2. Acquisition time increased with patient age both with the OCT1 and the OCT2 device. Most likely this is due to worse fixation and less endurance of older patients. Even in older patients, the acquisition duration with OCT2 is markedly lower than the acquisition time in younger age with OCT1. Considering the image quality of OCT2, Heidelberg Engineering states a better SNR and less signal roll-off which means the signal loss in anatomical deeper structures.

Resolution of the images should be similar to the OCT1 device. In our study, we found a significantly higher SNR of the OCT2 images. This is in line with findings of other OCT2 studies. Matteo et al. reported a more detailed imaging of the vitreoretinal interface, the retina, and the choroid by OCT2 technology.^11^ Despite the difference of the SNR, we found a good agreement of the RNFL thickness measurements of OCT1 and OCT2 which is in contrast to other similar studies. Vizzeri et al. reported a positive linear relationship between signal strength and RNFL thickness using Stratus OCT, but mean signal strength of the images was lower and relative differences between the analyzed images were higher compared to our study.^12^ In our study, we found smaller segmentation errors both in OCT1 and in OCT2 scans in a similar frequency. We had to exclude a small number of incomplete or incorrect scans which did not allow further analysis. Incorrect or incomplete scans occurred in patients with advanced glaucoma damage or with optic discs that are typically difficult to image such as tilted discs, microdiscs, macrodiscs, or peripapillary atrophy.

Both OCT1 and OCT2 use a specialized APS which refers to the fovea and the BMO center as anatomic landmarks. BMO and RNFL scans are then aligned to the fovea–disc axis, a constructed line between the fovea and the BMO center. This so-called FoDi alignment enables precise follow-up examinations, in comparison with reference data and an image analysis independent from, e.g., head tilt of the patient. The precision of the APS is based on an accurate determination of the BMO center and the fovea position. Valverde-Megías et al. found that an improper location of the fovea when using the FoDi alignment of Spectralis OCT leads to significant deviation of sectoral RNFL thickness measurements.^13^ In our study, we found a similar precision regarding the BMO center positioning of OCT1 and OCT2. In line with this, we found a good agreement between OCT1 and OCT2 regarding BMO-MRW- and RNFL-thickness measurements over the whole range of the mean values. This is also relevant for follow-up examinations which can be performed with an OCT2 device based on OCT1 examinations. Data compatibility allows the Spectralis software to generate these device comprehensive analyses.

## CONCLUSION

In conclusion, OCT2 technology can improve diagnostic imaging in glaucoma. With reduced acquisition time, the imaging process is easier for both the patient and the examiner. Image quality also appears to be better using OCT2 technology.
